# ﻿Pseudoscorpions (Arachnida, Pseudoscorpiones) from French Polynesia with first species records and description of new species

**DOI:** 10.3897/zookeys.1192.111308

**Published:** 2024-02-19

**Authors:** Katarína Krajčovičová, Thibault Ramage, Frédéric A. Jacq, Jana Christophoryová

**Affiliations:** 1 Bratislavské regionálne ochranárske združenie – BROZ, Na Riviére 7/a, 841 04, Bratislava, Slovakia Bratislavské regionálne ochranárske združenie – BROZ Bratislava Slovakia; 2 14 impasse Jeanne Dieulafoy, 29900, Concarneau, France Unaffiliated Concarneau France; 3 BP 41 405 Faretony, 98713 Papeete, Tahiti, French Polynesia Unaffiliated Tahiti French Polynesia; 4 Department of Zoology, Faculty of Natural Sciences, Comenius University, Mlynská dolina, Ilkovičova 6, 842 15, Bratislava, Slovakia Comenius University Bratislava Slovakia

**Keywords:** Endemism, insular fauna, Oceania, Society Islands, taxonomy

## Abstract

A new species *Olpiumcaputi***sp. nov.** from Tahiti is described here based on external characters. This is the first record of the family Olpiidae Banks, 1895 from French Polynesia. Additionally, the genus *Paratemnoides* Harvey, 1991 is recorded from French Polynesia for the first time with the full description of new-found specimens of *Paratemnoidesassimilis* (Beier, 1932). New localities of *Geogarypuslongidigitatus* (Rainbow, 1897) are added. An identification key to pseudoscorpions of French Polynesia is provided.

## ﻿Introduction

The Pacific Ocean contains about 25,000 islands, which have various geological origins such as continental fragments or volcanic hot-spots. Most of these islands are very distant from continents, and the most remote islands are northern and eastern Polynesia, in the Hawaiian Islands and French Polynesia (Dupon et al. 1993; Gillespie 2002; Gillespie et al. 2008). French Polynesia consists of 118 islands and atolls spread over 5 million km^2^ with a total land area of approximately 3660 km^2^. The islands form five archipelagos: Austral Islands, Gambier Islands, Marquesas Islands, Society Islands, and Tuamotu Islands (Gillespie et al. 2008). Biologists have been attracted to these regions since the 18^th^ century, but French Polynesia, by comparison to the Hawaiian Islands, has received much less attention, especially since the 1930s (Gillespie et al. 2008; Ramage 2017).

A phenomenon called taxonomic disharmony (Roderick and Gillespie 2016) can be observed in the arachnids of French Polynesia. No Amblypygi, Opiliones, Palpigradi, Ricinulei, Solifugae, or Thelyphonida are reported from the islands until now (Ramage 2017; WPC 2022). On the other hand, some of the arachnids of French Polynesia are represented by a high degree of endemism. Araneae of French Polynesia includes 113 species, of which 49 are endemic. The highest number of endemic forms occur in the families Salticidae and Tetragnathidae (Ramage 2017). A total of 248 species of Acari are known in French Polynesia. Most of the species belong to Sarcoptiformes of which 59 are endemic (Ramage 2017; WSC 2023). Two species of Scorpiones are reported from French Polynesia, the pantropical *Isometrusmaculatus* (De Geer, 1778) and *Liochelesaustralasiae* (Fabricius, 1775), which is widely distributed in Asia and the Pacific (Vaucel et al. 2022; Rein 2023). Recently one species of Schizomida has been discovered in French Polynesia. *Zomusbagnallii* (Jackson, 1908) has been collected in the Society Islands (Bora Bora, Huahine, Raiatea, Tahiti, and Tetiaroa) and Tuamotu archipelago (Anaa) (J. Cokendolpher pers. comm.; unpublished data).

Pseudoscorpions on these remote islands have received only a little interest. Contributions to the knowledge of pseudoscorpions of French Polynesia date back to the 1930s and are associated with the Pacific Entomological Survey (Chamberlin 1938, 1939a, 1939b). Since then, the French Polynesian pseudoscorpion fauna has been thought to be comprised of four species in four genera divided into three families (WPC 2022). The first record from French Polynesia was of *Americherneskanaka* (Chamberlin, 1938), which was described from Ua Pou in the Marquesas Islands and collected on Mount Tekohepu in dead stipes of *Cyathea* sp. (Chamberlin 1938). The record of *A.kanaka* in Chamberlin (1938) lacks a description of the species, which was given later by Chamberlin (1939b). The species’ description is based on a single male specimen that was originally classified in the genus *Lamprochernes* Tömösváry, 1883 (Chamberlin 1938, 1939b). Harvey (1990) transferred the species to the genus *Americhernes* Muchmore, 1976 based on the following characters: leg IV with four tactile setae, trichobothrium *it* farther from fingertip than the distance between *isb* and *ist.* Several specimens of *Haplochernesfunafutensis* (With, 1907) were collected on pandanus and *Taliparititiliaceum* (L.) Fryxell, 2001 trunks on Tahiti, Society Island (Chamberlin 1939a). Chamberlin (1939b) recorded the presence of *Oratemnussamoanus* Beier, 1932 on two neighbouring Marquesas islands, Eiao and Hatuta’a. The specimens of *O.samoanus* were found in dead wood, under bark, and under stones (Chamberlin 1939a). *Geogarypuslongidigitatus* (Rainbow, 1897) was reported and described as *Geogarypusmarquesianus* Chamberlin, 1939 from French Polynesia in the first place (Chamberlin 1939b). It was later synonymised and reported from several islands in the Marquesas, Society, and Tuamotu archipelagos (Harvey 2000; WPC 2022).

During surveys led by two of the authors (TR and FJ) in French Polynesia between 2017 and 2020, a few pseudoscorpion specimens were collected on Huahine and Tahiti in the Society Islands. These few specimens include a new species described as *Olpiumcaputi* sp. nov. and another species, *Paratemnoidesassimilis* (Beier, 1932), which is a new record and redescribed here based on well-conserved material.

## ﻿Materials and methods

The samples from Motuhionoa on Huahine were collected as part of an environmental diagnostic for the French Polynesian Agricultural Service, and those from Mount Marau on Tahiti as part of a large-scale survey of the arthropods of Society Islands led by two of the authors (TR and FJ).

All specimens were immersed in lactic acid for clearing and studied on temporary slide mounts. After the study, they were rinsed in water and returned to 75% ethanol.

Morphological and morphometric analyses were performed using a Leica DM1000 compound microscope with an ICC50 camera module (LAS EZ application v. 1.8.0). Measurements were taken from digital images using the AxioVision 40LE application. Digital photographs (Fig. 2) were taken using a Canon EOS 5D Mark II camera attached to a Zeiss Axio Zoom V16 stereomicroscope. Image stacks were produced manually, combined using Zerene Stacker software, and subsequently edited in Adobe Photoshop CC. Terminology follows Chamberlin (1931), Harvey (1992), and Judson (2007).

All specimens presented in this paper are deposited in the zoological collections of the Naturhistorisches Museum Wien, Austria (NHMW). For proper identification, specimens of *Paratemnoidesassimilis* (Beier, 1932) were compared with *Paratemnoides* specimens deposited in NHMW.

### ﻿Abbreviations

Setae on chelicera: ***bs***–basal, ***es***–exterior, ***gls***–galeal, ***is***–interior, ***ls***–laminal, ***sbs***–subbasal.

Trichobothria of moveable chelal finger: ***b***–basal, ***sb***–subbasal , ***st***–subterminal, ***t***–terminal; trichobothria of fixed chelal finger: ***eb***–exterior basal, ***esb***–exterior subbasal, ***est***–exterior subterminal, ***et***–exterior terminal, ***ib***–interior basal, ***isb***–interior subbasal, ***ist***–interior subterminal, ***it***–interior terminal; ***pc***–coupled sensillum.

## ﻿Results

### ﻿Taxonomy

**Family Atemnidae Kishida, 1929**

**Genus *Paratemnoides* Harvey, 1991**

#### ﻿ Paratemnoides assimilis

Taxon classificationAnimaliaPseudoscorpionesAtemnidae

(Beier, 1932)

3E097E4C-93EF-5759-B5F2-C4802F210AD1

Figs 2A , 3

##### Materials examined

**Figure 1.**
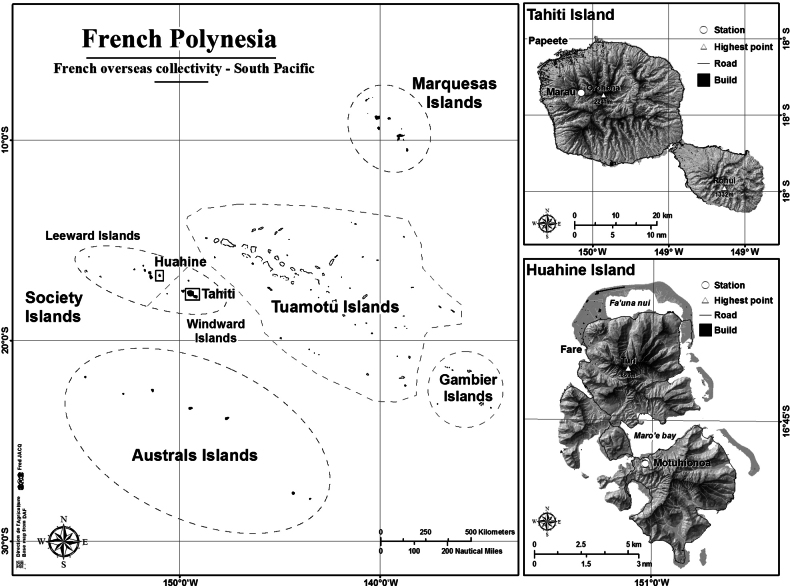
Map of archipelagos of French Polynesia with details of Tahiti and Huahine islands with marked studied localities.

**(Fig. 1).** French Polynesia • 2 ♂♂, 5 ♀♀, 1 tritonymph, 1 deutonymph; Huahine, Motuhionoa [16°46'16"N, 151°00'14"W]; 82 m a.s.l.; 06 Nov. 2020; F. Jacq leg.; decaying *Falcatariamoluccana* trunk; NHMW 29976. • 1 ♀; Huahine, Motuhionoa [16°46'11"N, 151°00'10"W]; 32 m a.s.l.; 06 Nov. 2020; F. Jacq leg.; Malaise trap; NHMW 29977.

##### Description.

♂ (♀) (Figs 2A, 3).

**Figure 2.**
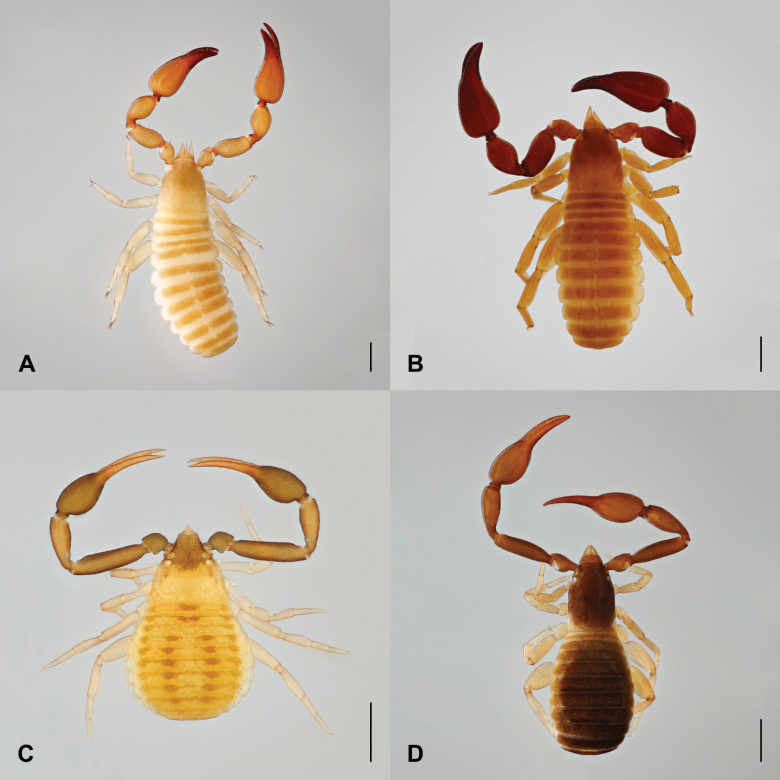
**A***Paratemnoidesassimilis*, female **B***Paratemnoidespallidus*, male **C***Geogarypuslongidigitatus*, male **D***Olpiumcaputi* sp. nov., female. Scale bars: 0.5 mm.

**Figure 3.**
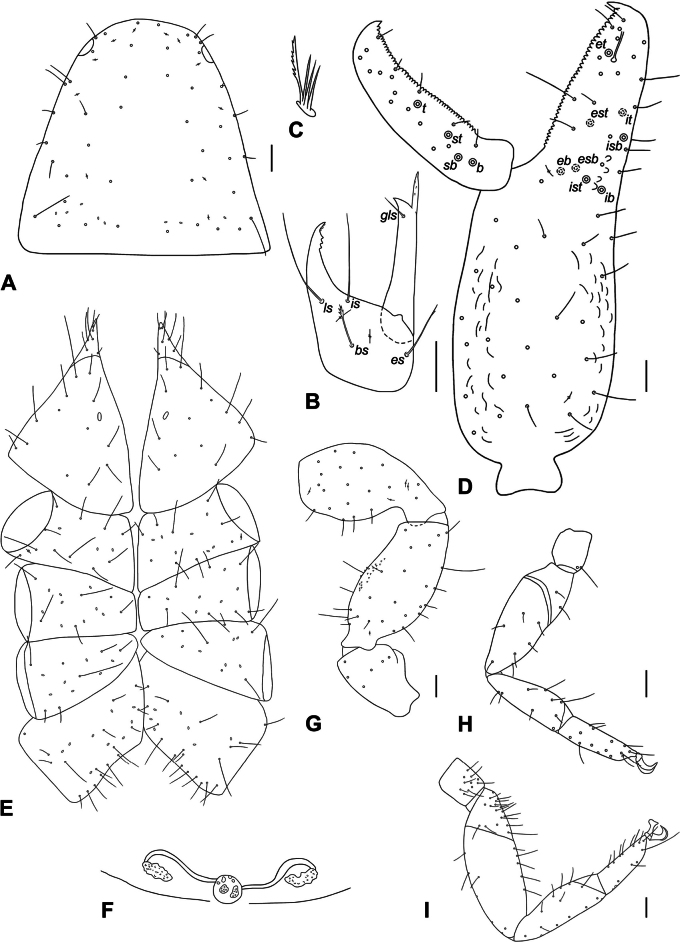
*Paratemnoidesassimilis***A** carapace, dorsal view **B** chelicera with setae pattern, dorsal view **C** rallum **D** palpal chela, dorsal view, showing trichobothriotaxy, teeth and venom apparatus **E** coxal area, ventral view **F** genital area **G** pedipalp, dorsal view (trochanter, femur, and patella) **H** leg I, lateral view **I** leg IV, lateral view. Scale bars: 0.1 mm.

***Carapace*** (Fig. 3A). Carapace 0.96 (0.95) × longer than broad, conically narrowed; epistome absent; with 2 distinct eye spots; smooth, without transverse furrows; anterior half brown distinct darker than posterior half; with 49 (44) acuminate setae apically with a dentition, 8 (9) setae on posterior margin; for lyrifissures see Fig. 3A. ***Chelicera*** (Fig. 3B, C) . Chelicera 2.33 (2.47) × longer than broad; 4 setae on hand, *sbs* absent, *bs* and *es* denticulate; moveable finger with 1 short seta; 2 slit-like lyrifissures on hand; galea long with 5 rami (Fig. 3B); serrula exterior with 22 blades; rallum consisting of 4 blades, distal one long and serrated (Fig. 3C). ***Pedipalps*** (Fig. 3D, G). Pedipalps smooth, only anterior face of femur with minute denticles (Fig. 3G), trochanter and femur lighter than chela (Fig. 2A). Trochanter 1.59×, femur 2.16 (2.19) ×, patella 1.91 (1.94) ×, chela 2.59 (2.63) ×, hand with pedicel 1.67 (1.69) × longer than broad. Venom apparatus present only in fixed finger terminating in nodus ramosus slightly proximal to trichobothria *et* (Fig. 3D). Fixed chelal finger with 8 trichobothria, moveable chelal finger with 4 trichobothria. Fixed chelal finger with 39 small marginal teeth; moveable chelal finger with 42 (43) small marginal teeth (Fig. 3D). Trichobothia *eb* and *esb* adjacent and located basally; *est* midway between *esb* and *et*; *st* closer to *sb* than to *t.* For a complete trichobothrial pattern see Fig. 3D. ***Coxae*** (Fig. 3E). Coxae smooth, all setae acuminate or with fine dentition apically; manducatory processes with 6 (5) setae; palpal coxae with 13–14 (16–17) setae; pedal coxae I–IV chaetotaxy: 9–11 (8–10): 6–8 (9): 6–7 (7): 17–19 (15–17); for lyrifissures see Fig. 3E. ***Abdomen*** (Fig. 2A). Tergites I–III undivided, IV–V partly divided, VI–XI with fine division; sternites IV–XI divided. All setae acuminate or with fine dentition apically. ***Tergal chaetotaxy* I–XI**: 8: 8: 8: 4–5: 6–7 (6–7): 6 (6–7): 6 (6–7): 6 (7): 7: 9: 5. ***Sternal chaetotaxy* IV–XI**: 4: 5–7 (7): 6 (7–8): 7 (8): 8 (7–8): 9 (7): 7: 5. ***Genital area* II–III**. Short acuminate setae [slit-like lyrifissures]: 15 [2] (8 [4]): 6 [4] (4 [2]). ***Genital area*** (Fig. 3F). Male rod Y shaped, female with several cribriform plates externally (Fig. 3F). ***Leg* I** (Fig. 3H). Trochanter 1.21×, femur I 1.25 (1.26) ×, femur II 2.86 (2.63) ×, tibia 2.77×, tarsus 3.22 (3.33) × deeper than broad. ***Leg* IV** (Fig. 3I). Trochanter 1.44×, femoropatella 3.15 (3.23) ×, tibia 3.18 (3.29) ×, tarsus 2.77 (2.69) × deeper than broad. Leg IV with 1 tactile seta basally on tarsus (Fig. 3I). Claws simple, arolium slightly shorter than claws (Fig. 3H, I).

##### Measurements

**(in mm, length/width or, for legs, length/depth).** ♂ (♀). Body length 2.98 (3.38). Pedipalps: trochanter 0.43/0.27, femur 0.69 (0.70)/0.32, patella 0.67 (0.68)/0.35, chela 1.27 (1.29)/0.49, hand with pedicel 0.82 (0.83)/0.49, hand without pedicel 0.72 (0.73), fixed finger 0.62 (0.63). Chelicera 0.35 (0.37)/0.15, moveable finger 0.28. Carapace 0.85 (0.88)/0.89 (0.93). Leg I: trochanter 0.17/0.14, femur I 0.25 (0.24)/0.20 (0.19), femur II 0.40 (0.42)/0.14 (0.16), tibia 0.36/0.13, tarsus 0.29 (0.30)/0.09. Leg IV: trochanter 0.23/0.16, femoropatella 0.82 (0.84)/0.26, tibia 0.54/0.17, tarsus 0.36 (0.35)/0.13.

##### Identification.

*Paratemnoidesassimilis*is most similar to *P.pallidus* (Balzan, 1892) as both possess similar proportions of the palpal segments (femur 0.62–0.83 mm long/2.10–2.30× longer than broad, patella 0.59–0.78 mm long/1.80–1.90× longer than broad, chela 1.27–1.44 mm long/2.40–2.70× longer than broad, finger 0.47–0.63 mm long), minute denticles on the palpal segments while other body segments are smooth, and a carapace without transverse furrows. They differ by the presence of minute denticles on different segments of the pedipalps; in *P.assimilis* denticles are present on the anterior margin of the palpal femur, but with other palpal segments smooth, but in *P.pallidus* denticles are present on femur as well as on patella (Beier 1932a, 1932b; Mahnert 1978a; Harvey 1988). Measurements of the palpal hand with pedicel also differ (*P.assimilis* 0.62–0.83 mm long vs *P.pallidus* 0.80–1.06 mm long) (Mahnert 1978a; Harvey 1988).

##### Remarks.

New-found specimens of *P.assimilis* were compared with selected *Paratemnoides* species deposited in NHMW: *P.assimilis* [NHMW-Zoo-AR 25115, NHMW-Zoo-AR 25124]; *P.ceylonicus* Beier, 1932 [NHMW-Zoo-AR 25064, NHMW-Zoo-AR 25065]; *P.curtulus* (Redikorzev, 1938) [NHMW-Zoo-AR 25117]; *P.laosanus* (Beier, 1951) [NHMW-Zoo-AR 25073]; *P.pallidus* [NHMW-Zoo-AR 25090, NHMW-Zoo-AR 25125]; and *P.salomonis* (Beier, 1935) [NHMW-Zoo-AR 25110]. As mentioned by Harvey (1988), the identification keys to *Paratemnoides* species by Beier (1932a, 1932b) are based generally on the size or thickness of palpal segments and legs. Harvey (1988) applied the character of measurements of leg segments as distinguishing ones for *P.assimilis* and *P.ceylonicus*. Specimens of species mentioned above were examined and compared in this study. Leg segments were measured but the values completely overlapped in all examined species. Considering these results and the fact that the *Paratemnoides* species descriptions are generally not sufficient, a revision of this genus is necessary to clearly set species boundaries.

Currently, *P.ceylonicus*is one of the synonyms of *P.pallidus* (Fig. 2B) (Klausen 2005; WPC 2022). The current synonymy of the two species was justified by no significant difference between *P.ceylonicus* and *P.pallidus* in palpal chela measurements (Klausen 2005). Beier (1932a, 1932b, 1973) supported the existence of *P.ceylonicus* by the presence of minute denticles on the anterior margin of the palpal femur while other palpal segments are smooth.

All examined specimens of *P.ceylonicus* deposited in NHMW possess distinct granulation present on palpal femur as well as on patella just like in *P.pallidus*. The present study supports the synonymization of *P.ceylonicus* with *P.pallidus* suggested by Klausen (2005).

**Family Geogarypidae Chamberlin, 1930**

**Genus *Geogarypus* Chamberlin, 1930**

#### ﻿ Geogarypus longidigitatus

Taxon classificationAnimaliaPseudoscorpionesGeogarypidae

(Rainbow, 1897)

820D388D-91ED-5BBF-A191-E2950A72B0EE

Fig. 2C

##### Materials examined

**(Fig. 1).** French Polynesia • 1 ♂; Huahine, Motuhionoa [16°46'15"N, 151°00'12"W]; 61 m a.s.l.; 06 Nov. 2020; F. Jacq leg.; *Mangi­fera indica* and *Taliparititiliaceum* forest, leaf litter sifting; NHMW 29978. • 1 ♂, 1 deutonymph; Huahine, Motuhionoa [16°46'16"N, 151°00'12"W]; 79 m a.s.l.; 06 Nov. 2020; F. Jacq leg.; *Taliparititiliaceum* forest, leaf-litter sifting; NHMW 29979.

##### Measurements

**(in mm, length/width).** ♂. Body length 1.57. Pedipalp: trochanter 0.22/0.16, femur 0.55/0.13, patella 0.40/0.15, chela 0.91/0.23, hand with pedicel 0.44/0.23, fixed finger length 0.51. Carapace 0.53–0.55/0.64.

##### Identification.

*Geogarypuslongidigitatus*is remarkably similar to *G.ocellatus* Mahnert, 1978, as both possess the same pattern of carapace coloration, but the palpal patella and chela of *G.ocellatus* are more slender than in *G.longidigitatus* (e.g. patella and chela: *G.longidigitatus* 2.5–2.6× longer than broad and 3.5–4.2× longer than broad vs. *G.ocellatus* 3.0–3.3× longer than broad and 4.1–4.5× longer than broad) (Mahnert 1978b; Harvey 2000). See Harvey (2000) for the complete redescription of *G.longidigitatus* and diagnosis of other geogarypid species. Newly described geogarypids found in the Asian-Australian-Pacific regions differ from *G.longidigitatus* as follows: *G.muchmorei* Novák & Harvey, 2018 differs by its larger area of brown coloration on the carapace and the swollen margin of the chelal hand; *G.klarae* Novák & Harvey, 2018 differs by having a white palpal trochanter and strongly curved teeth on the fixed chelal finger (Novák and Harvey 2018); *G.plusculus* Cullen & Harvey, 2021 and *G.facetus* Cullen & Harvey, 2021 differ by the patchy coloration of the carapace and brighter palpal trochanter and femur (Cullen and Harvey 2021).

##### Remarks.

The species is widely distributed in the Indo-Pacific region (Novák and Harvey 2018; WPC 2022). Harvey (2000) assumed that the wide distribution of the species is also due to human activities.

**Family Olpiidae Banks, 1895**

**Genus *Olpium* L. Koch, 1873**

#### ﻿ Olpium caputi

Taxon classificationAnimaliaPseudoscorpionesOlpiidae

Krajčovičová & Christophoryová sp. nov.

78850127-A872-5546-949E-74C993CB0136

https://zoobank.org/A27FF8CF-E164-4A69-8787-FE60F61300EB

Figs 2D , 4

##### Material examined

(Fig. 1). ***Holotype***: French Polynesia • 1 ♀; Tahiti, Mont Marau Summit [17°36'52"N, 149°31'45"W]; 1450 m a.s.l.; 01 Sept. 2017; F.A. Jacq & T. Ramage leg.; sifting of epiphyte moss on *Pterophyllaparviflora* (G.Forst.) Pillon & H.C.Hopkins; NHMW 29980.

##### Etymology.

The species’ epithet is a patronym honouring Zuzana Čaputová, the Slovak President. As a female leader, she expresses clear attitudes and supports women as well as scientists. In this manner, we would like to pay tribute to her.

##### Diagnosis.

*Olpiumcaputi* sp. nov. is most similar to *O.afghanicum* Beier, 1952 and *O.philippinum* Beier, 1967, as all possess a dark brown carapace, pedipalps, and abdomen, and with carapace and abdomen being darker than palpal segments, a carapace without transverse furrows, and similar proportions of the palpal segments (e.g. patella 2.80–3.30× longer than broad, chela with pedicel 3.40–4.00× longer than broad and chelal finger 0.60–0.63 mm long) (Beier 1952, 1967). *Olpiumcaputi* sp. nov. differs from *O.afghanicum* in having smooth chelal hands and two enlarged setae present on the palpal femur, while in *O.afghanicum* the the chelal hands possess mediodistal dense granulation and only one enlarged seta is present on palpal femur (Beier 1952). *Olpiumcaputi* sp. nov. differs from *O.philippinum* in having all palpal segments smooth, while *O.philippinum* possesses sparse mediodistal granulation on the palpal trochanter and hand. In addition, the palpal femur of *O.caputi* sp. nov. is more slender than in *O.philippinum* (e.g. palpal femur in *O.caputi* sp. nov. 4.11× longer than broad vs that of *O.philippinum* 3.20–3.30× longer than broad) (Beier 1967).

##### Description.

**Figure 4.**
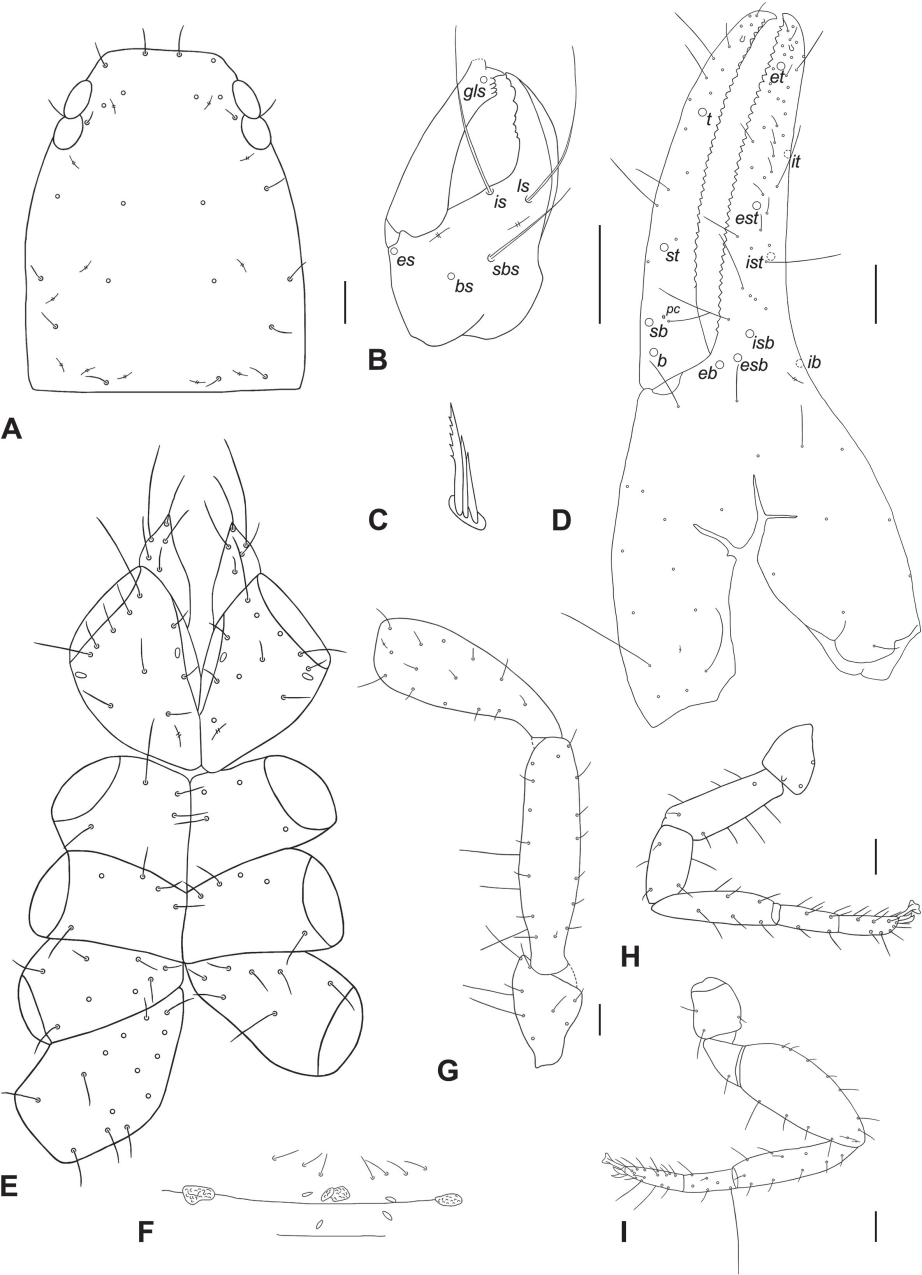
*Olpiumcaputi* sp. nov. **A** carapace, dorsal view **B** chelicera with setae pattern, dorsal view **C** rallum **D** palpal chela, dorsal view, showing trichobothriotaxy, teeth and venom apparatus **E** coxal area, ventral view **F** genital area **G** pedipalp, dorsal view (trochanter, femur, and patella) **H** leg I, lateral view **I** leg IV, lateral view. Scale bars: 0.1 mm.

♀ (Figs 2D, 4). Integument pigmented; carapace, pedipalps, and abdomen dark brown; carapace and abdomen slightly darker than palpal segments; tergites I–II whitish, following tergites brown, markedly darker (Fig. 2D). ***Carapace*** (Fig. 4A). Carapace 1.33× longer than broad, rectangular without transverse furrows; 4 eyes, the anterior ones with very convex lens, both pairs with tapetum; 25 thin setae, of which 4 anterior and 3 posterior; with 12 lyrifissures. ***Chelicera*** (Fig. 4B, C). Chelicera 2.08× longer than broad, palm with 5 acuminate setae; fixed finger with 7 teeth; moveable finger with 1 subdistal seta, galea broken apically (Fig. 4B), rallum with 3 blades, distal one serrated (Fig. 4C), serrula exterior with 17 blades. ***Pedipalps*** (Figs 2D, 4D, G). Pedipalps smooth (Figs 2D, 4G). Trochanter 1.95×, femur 4.11×, patella 2.83×, chela 3.74×, hand with pedicel 1.82× longer than broad. Femur dorsal with 2 elongate setae without enlarged alveoli (Fig. 4G). Venom apparatus very short present in both fixed and moveable fingers terminating in nodus ramosus distal to trichobothrium *et* on fixed finger (Fig. 4D). Fixed chelal finger with 8 trichobothria, moveable chelal finger with 4 trichobothria. Fixed chelal finger with 41 slightly reclined and pointed teeth; moveable chelal finger with 35 small marginal teeth (Fig. 4D). A coupled sensillum (*pc*) closer to *sb* than to *st.* Trichobothria *eb*, *esb*, *ib*, *isb* located on the base of the fixed finger; *est* closer to *ist* than to *it*; *b* and *sb* located on the base of the moveable finger; *st* closer to *sb* than to *t.* For a complete trichobothrial pattern see Fig. 4D. ***Coxae*** (Fig. 4E). Coxae smooth, all setae acuminate; manducatory processes with 5 setae; palpal coxae with 10–11 setae; pedal coxae I–IV chaetotaxy: 4: 5: 7–8: 14 (1 damaged); for lyrifissures see Fig. 4E. ***Abdomen*** (Fig. 2D). Tergites longitudinally not divided. Pleural membrane longitudinally striate. ***Tergal chaetotaxy* I–X**: 2: 4: 5: 4: 4: 4: 4: 4: 6: 10. ***Chaetotaxy of sternites* II–X**: 7: 4: 4: 6: 8: 6: 6: 9: 10. Genital area very simple with marginal row of 7 acuminate setae on posterior operculum; one pair of lateral cribriform plates and one pair of medial cribriform plates next to each other as on Fig. 4F. ***Leg* I** (Fig. 4H). Trochanter 1.38×, femur 3.00×, patella 1.91×, tibia 4.43×, tarsus I 3.40×, tarsus II 3.00× deeper than broad. ***Leg* IV** (Fig. 4I). Trochanter 1.53×, femoropatella 2.87×, tibia 4.36×, tarsus I 2.86×, tarsus II 3.33× deeper than broad. Leg IV with a long tactile seta basally on tarsus I (Fig. 4I). Claws simple, arolium significantly longer than claws (Fig. 4H–I).

##### Measurements

**(in mm, length/width or, for legs, length/depth).** ♀. Body length 2.28. Pedipalps: trochanter 0.39/0.20, femur 0.74/0.18, patella 0.65/0.23, chela 1.27/0.34, hand with pedicel 0.62/0.34, hand without pedicel 0.54, moveable finger 0.67. Chelicera 0.27/0.13, moveable finger 0.17. Carapace 0.80/0.60. Leg I: trochanter 0.18/0.13, femur 0.30/0.10, patella 0.21/0.11, tibia 0.31/0.07, tarsus I 0.17/0.05, tarsus II 0.15/0.05. Leg IV: trochanter 0.26/0.17, femoropatella 0.66/0.23, tibia 0.48/0.11, tarsus I 0.20/0.07, tarsus II 0.20/0.06.

##### Distribution and ecology.

Currently, this species is known only from the type locality in Tahiti, French Polynesia. The specimen was collected by sifting from epiphyte moss.

##### Remarks.

Dashdamirov and Schawaller (1993) questioned the affiliation of *O.afghanicum* within the genus *Olpium* L. Koch, 1873 based on the following characters: nodus ramosus is distal of trichobothrium *et*, tarsus I is longer than tarsus II, the first tergite and posterior margin of carapace bear four setae. As mentioned in Murthy and Ananthakrishnan (1977), the length of nodus ramosus, given by Hoff (1964) for Olpinii with *Olpium* as the type genus, cannot be satisfactorily used to distinguish *Olpium* from other genera. As explained by Harvey and Leng (2008), almost all Olpiinae Banks, 1895 possess short venom ducts not reaching *et* on the fixed chelal finger. The redescriptions of *Olpiumpallipes* (Lucas, 1849) and *Olpiumkochi* Simon, 1881 show the variability in setae number on the posterior margin of carapace, both species bear 4–5 setae on it (Heurtault 1979; Mahnert 1981). New described *O.caputi* sp. nov. possesses a very short venom apparatus terminating in nodus ramosus distal to trichobothrium *et* and three setae are present on the posterior margin of carapace.

### ﻿Identification key to pseudoscorpion species from French Polynesia

**Table:** 

1	Carapace subtriangular, brown in anterior half, posterior half creamy white; eyes situated away from anterior margin of carapace; palpal segments brown: femur 0.46–0.81 mm long; chela with pedicel 0.82–1.24 mm long; moveable finger 0.47–0.70 mm long; anal plate located between tergite and sternite XI	***Geogarypuslongidigitatus***
–	Carapace subrectangular; eyes situated near anterior margin of carapace	**2**
2	Spermatheca absent; all body segments smooth without granulation; carapace and abdomen darker than palpal segments; tergites I–II whitish; carapace without transverse furrows; 4 eyes, the anterior ones with very convex lens; palpal femur 4.11× longer than broad and in basal half with 2 trichobothria (enlarged setae); femur I of leg I as long as femur II or longer	***Olpiumcaputi* sp. nov**.
–	Spermatheca present; palpal femur without trichobothria; male sternites without discrete patches of sensory organs and without coxal sacks or ram’s horn organs	**3**
3	Venom apparatus present in moveable finger only; chelal fingers normally with at least one accessory tooth; carapace with indistinct eye spots	**4**
–	Venom apparatus present in fixed finger only; chelal fingers without accessory teeth; carapace with 2 distinct eye spots	**5**
4	Small species; palpal femur 0.42–0.44 mm long; palpal chela with pedicel 0.72 mm long; chelal fingers 0.36 mm long; venom apparatus terminating in nodus ramosus at the level of trichobothrium *t*; female spermatheca consisting of 2 separate curved tubes terminating in cylindrical sacks	***Americherneskanaka***
–	Large species; palpal femur 0.59–0.74 mm long; palpal chela with pedicel 1.09–1.34 mm long; chelal fingers 0.52–0.67 mm long; venom apparatus terminating in nodus ramosus submedialy between trichobothria *t* and *st*; female spermatheca unpaired and T-shaped	***Haplochernesfunafutensis***
5	Trichobothrium *it* of fixed chelal finger distant from the fingertip at most as distance between *ist* and *isb*; venom apparatus terminating in nodus ramosus slightly distal to trichobothria *est*; palpal segments smooth, except for small and scattered granulations exteriorly on trochanter, interiorly on femur and patella and at the base of chelal fingers; chelal fingers shorter than the width of chelal hand	***Oratemnussamoanus***
–	Trichobothrium *it* of fixed chelal finger distant from the fingertip further than distance between *ist* and *isb*; venom apparatus terminating in nodus ramosus slightly proximal to trichobothria *et*; palpal segments smooth, only anterior face of femur with minute denticles; chelal fingers longer than the width of chelal hand	***Paratemnoidesassimilis***

## ﻿Discussion

Much of the Pacific Basin was colonized by animals primarily from New Guinea and adjacent areas via over-water dispersal. Small islands were “stepping stones”, facilitating dispersal across the Pacific (Miller 1996). Munroe (1996) showed that there is a progressive decrease in the number of founding stocks and an increase in the proportion of radiating speciation with distance from Papuan source areas, also known as the “radiation zone” (MacArthur and Wilson 1967). This, and the taxonomic disharmony it induced, led to many free ecological niches and so a strong endemism developed. Ramage (2017) indicated that 61% of French Polynesia native terrestrial arthropods are endemic, which is similar to the flora (62%) and avifauna (64%), but far less than the exceptional level of endemism of the snail fauna (95%).

Two pseudoscorpion species are known to occur only in French Polynesia, *Americherneskanaka* (WPC 2022) and the newly described *Olpiumcaputi* sp. nov. They could be considered endemic, but, as pointed out by Chamberlin (1939a), the single island endemism of some pseudoscorpion species is doubtful. Our knowledge about pseudoscorpions in Oceania is still very limited. There are natural ways in which they are distributed such as phoresy and introductions via transport must also be taken into account. Even if the pseudoscorpions were not explicitly undertaking phoresy during the research, it must be considered that some of the populations may have become established after transportation on an aerial host. Pseudoscorpions may also naturally arrive in French Polynesia under the bark of floating trunks or transported by Austronesians with root vegetables, as they did with ants (Ramage 2014).

*Paratemnoidesassimilis* was originally described from the Philippines and later discovered on Java and Krakatau Islands (Harvey 1988; WPC 2022). It was collected from various habitats, such as under the bark of a dead tree, in vegetation, in litter, and inside a tent (Harvey 1988). Several specimens presented in the current study were found on the island of Huahine for the first time. The specimens were found in a decaying tree trunk and Malaise trap. *Geogarypuslongidigitatus* was originally described from Funafuti, one of the islands of Tuvalu (Rainbow 1897). *Geogarypuslongidigitatus*, with its numerous synonyms, is known to have an extremely wide distribution and is also found in various habitats such as in litter and soil, on decaying substrates, in vegetation (moss, fern, grass, epiphyte), under stones, on rock walls, under bark, and in the roadside bush with anthropochorous vegetation (Chamberlin 1939b; Harvey 2000). All specimens presented in this study were found for the first time on the island of Huahine and were collected by leaf-litter sifting.

## Supplementary Material

XML Treatment for Paratemnoides assimilis

XML Treatment for Geogarypus longidigitatus

XML Treatment for Olpium caputi

## ﻿Additional information

### Author ORCIDs

Katarína Krajčovičová https://orcid.org/0000-0003-1303-2434

Thibault Ramage https://orcid.org/0000-0001-5939-7098

Frédéric A. Jacq https://orcid.org/0000-0002-9177-6212

Jana Christophoryová https://orcid.org/0000-0002-3746-1367

### Data availability

All of the data that support the findings of this study are available in the main text.
